# Di-μ-azido-κ^4^ *N*:*N*-bis­({2-[(3-amino-2,2-dimethyl­prop­yl)imino­meth­yl]-6-meth­oxy­phenolato-1κ^3^ *N*,*N*′,*O* ^1^}copper(II))

**DOI:** 10.1107/S1600536812028954

**Published:** 2012-06-30

**Authors:** Akbar Ghaemi, Saeed Rayati, Kazem Fayyazi, Seik Weng Ng, Edward R. T. Tiekink

**Affiliations:** a Department of Chemistry, Saveh Branch, Islamic Azad University, Saveh, Iran; b Department of Chemistry, K. N. Toosi University of Technology, PO Box 16315-1618, Tehran, Iran; c Department of Chemistry, University of Malaya, 50603 Kuala Lumpur, Malaysia; d Chemistry Department and Faculty of Science, King Abdulaziz University, PO Box 80203 Jeddah, Saudi Arabia

## Abstract

The complete mol­ecule of the title complex, [Cu_2_(C_13_H_19_N_2_O_2_)_2_(N_3_)_2_], is generated by the application of a centre of inversion. The central Cu_2_N_2_ core is a rhombus as the μ_2_-azide ligands bridge in an asymmetric fashion. Each Cu^II^ atom is also coordinated by a monoanionic tridentate Schiff base ligand *via* the anti­cipated oxide O, imine N and amine N atoms. The resulting N_4_O coordination geometry is based on a square pyramid. No specific inter­molecular inter­actions are noted in the crystal packing, but the amine H atoms form intra­molecular N—H⋯O(oxide)/N(azide) hydrogen bonds.

## Related literature

For background to azido derivatives of tridentate Schiff base copper(II) structures, see: Adhikary & Koner (2010 ▶). For a related structure, see: Ghaemi *et al.* (2012 ▶). For additional structural analysis, see: Addison *et al.* (1984 ▶).
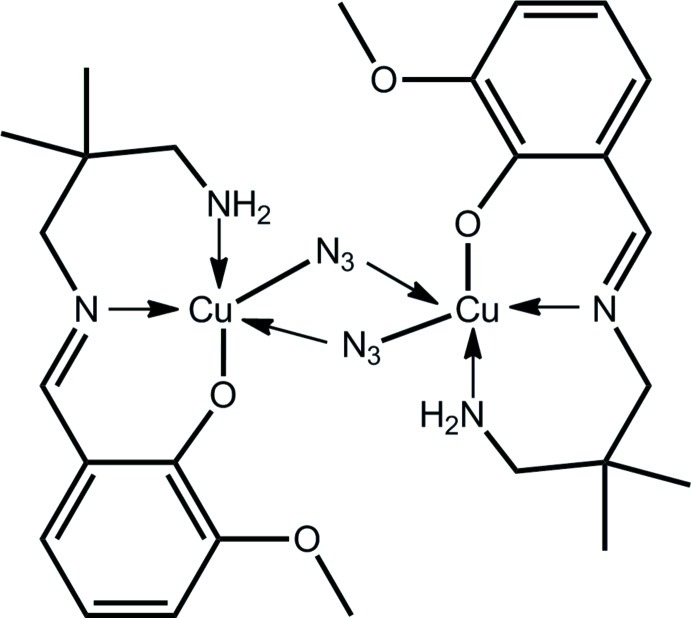


## Experimental

### 

#### Crystal data

[Cu_2_(C_13_H_19_N_2_O_2_)_2_(N_3_)_2_]
*M* *_r_* = 681.76
Monoclinic, 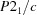

*a* = 9.1733 (5) Å
*b* = 12.2369 (5) Å
*c* = 13.0988 (6) Å
β = 98.203 (5)°
*V* = 1455.33 (12) Å^3^
*Z* = 2
Mo *K*α radiation
μ = 1.51 mm^−1^
*T* = 100 K
0.20 × 0.15 × 0.10 mm

#### Data collection

Agilent SuperNova Dual diffractometer with an Atlas detector
Absorption correction: multi-scan (*CrysAlis PRO*; Agilent, 2010 ▶) *T*_min_ = 0.674, *T*_max_ = 1.000
5747 measured reflections
3314 independent reflections
2628 reflections with *I* > 2σ(*I*)
*R*_int_ = 0.034

#### Refinement

*R*[*F* ^2^ > 2σ(*F* ^2^)] = 0.039
*wR*(*F* ^2^) = 0.105
*S* = 1.06
3314 reflections
198 parameters
2 restraints
H atoms treated by a mixture of independent and constrained refinement
Δρ_max_ = 0.54 e Å^−3^
Δρ_min_ = −0.52 e Å^−3^

### 

Data collection: *CrysAlis PRO* (Agilent, 2010 ▶); cell refinement: *CrysAlis PRO*; data reduction: *CrysAlis PRO*; program(s) used to solve structure: *SHELXS97* (Sheldrick, 2008 ▶); program(s) used to refine structure: *SHELXL97* (Sheldrick, 2008 ▶); molecular graphics: *ORTEP-3 for Windows* (Farrugia, 1997 ▶) and *DIAMOND* (Brandenburg, 2006 ▶); software used to prepare material for publication: *publCIF* (Westrip, 2010 ▶).

## Supplementary Material

Crystal structure: contains datablock(s) global, I. DOI: 10.1107/S1600536812028954/sj5249sup1.cif

Structure factors: contains datablock(s) I. DOI: 10.1107/S1600536812028954/sj5249Isup2.hkl

Additional supplementary materials:  crystallographic information; 3D view; checkCIF report

## Figures and Tables

**Table 1:** Selected bond lengths (Å)

Cu—O2	1.9047 (18)
Cu—N1	1.960 (2)
Cu—N2	2.001 (2)
Cu—N3	2.023 (2)
Cu—N3^i^	2.641 (2)

Symmetry code: (i) 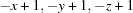.

**Table 2:** Hydrogen-bond geometry (Å, °)

*D*—H⋯*A*	*D*—H	H⋯*A*	*D*⋯*A*	*D*—H⋯*A*
N2—H1*N*⋯O2^i^	0.87 (1)	2.35 (2)	2.956 (3)	127 (2)
N2—H2*N*⋯N3	0.88 (1)	2.36 (3)	2.752 (3)	107 (3)

Symmetry code: (i) 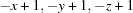.

**Fig. 1.**
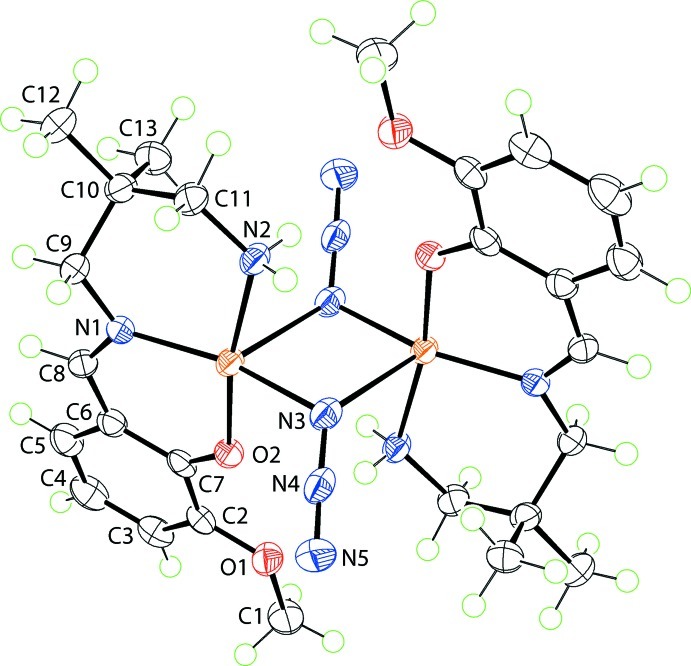
The molecular structure of (I) showing the atom-labelling scheme and displacement ellipsoids at the 70% probability level.

**Fig. 2.**
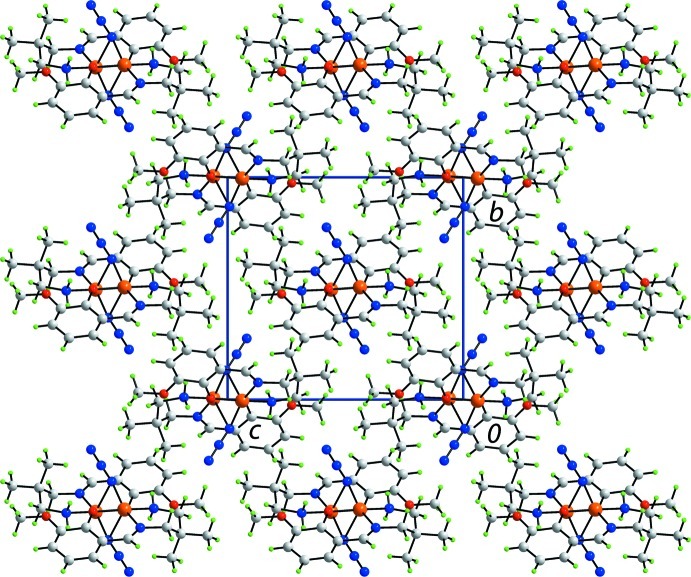
A view in projection down the a axis of the unit-cell contents of (I).

## supplementary crystallographic information

### Comment

Azido-bridged copper(II) complexes continue to attract attention in relation to investigations of small molecule activation of copper-containing proteins and for new magnetic materials (Adhikary & Koner, 2010). Recently, the crystal structure of a related Ni^II^ complex was described in which the Schiff base ligand was shown to coordinate in two distinct modes, *i.e*. a tridentate mode towards one Ni^II^ atom and in a pentadentate mode, bridging two Ni^II^ atoms (Ghaemi *et al.*, 2012).

In the centrosymmetric binuclear complex (I), Fig. 1, the Cu^II^ atoms are bridged by one end of each of two µ_2_-azido ligands to generate an Ni_2_N_2_ core with the shape of a rhombus as the bridge is asymmetric, Table 1. The coordination geometry for the Cu^II^ atom is completed by the oxido-O, imine-O and amino-N donor atoms derived from a tridentate uninegative Schiff base ligand. The N_4_O donor set defines a coordination geometry close to square pyramidal. This is quantified by the value of τ = 0.12 which compares to the τ values of 0.0 and 1.0 for ideal square pyramidal and trigonal bipyramidal geometries, respectively (Addison *et al.*, 1984). The configuration is stabilized by an intramolecular N—H···O(oxido) and *N*—*H*···*N*(azido) hydrogen bonds, Table 2. Globally, molecules stack in columns aligned along the *a* axis, Fig. 2, without specific intermolecular interactions between them.

### Experimental

A mixture of 2,2-dimethylpropylenediamine (0.234 g, 2.3 mmol) was added to a clear solution of Cu(NO_3_)_2_.3H_2_O (0.50 g, 2.07 mmol) dissolved in methanol (25 ml), which immediately produced an intense-blue solution. The solution was then heated to boiling and a methanolic solution of 2-hydroxy-3-methoxybenzaldehyde (0.273 g, 1.8 mmol) was added drop-wise over 2 h under refluxing conditions. Reflux was continued for another 45 min. Then an excess sodium azide (0.5 g, 7.7 mmol) dissolved in water (2 ml) was added. The precipitate was filtered and dissolved in methanol. Brown crystals were formed within a few days from the methanolic solution. Anal. Calc. for C_26_H_38_Cu_2_N_10_O_4_: C, 45.81; H, 5.62; N, 20.55. Found: C, 45.77; H, 5.57; N, 20.66%. IR (KBr) [ν, cm^-1^]: ν_as_(N_3_) 2035 *versus*, ν(C═N) 1621 s, ν(C═C) 1540 s, ν(C—O) 1224 m. *M*.pt: 476–478. Yield: 60%.

### Refinement

Carbon-bound H-atoms were placed in calculated positions [C—H = 0.95–0.99 Å, *U*_iso_(H) = 1.2–1.5*U*_eq_(C)] and were included in the refinement in the riding model approximation. The amino H-atoms were located from a difference map and refined with N—H = 0.88±0.01 and with *U_iso_*(H) = 1.2*U_eq_*(N).

### Crystal data

**Table:**

[Cu_2_(C_13_H_19_N_2_O_2_)_2_(N_3_)_2_]	*F*(000) = 708
*M**_r_* = 681.76	*D*_x_ = 1.556 Mg m^−^^3^
Monoclinic, *P*2_1_/*c*	Mo *K*α radiation, λ = 0.71073 Å
Hall symbol: -P 2ybc	Cell parameters from 2122 reflections
*a* = 9.1733 (5) Å	θ = 2.5–27.5°
*b* = 12.2369 (5) Å	µ = 1.51 mm^−^^1^
*c* = 13.0988 (6) Å	*T* = 100 K
β = 98.203 (5)°	Prism, brown
*V* = 1455.33 (12) Å^3^	0.20 × 0.15 × 0.10 mm
*Z* = 2	

### Data collection

**Table:**

Agilent SuperNova Dual diffractometer with an Atlas detector	3314 independent reflections
Radiation source: SuperNova (Mo) X-ray Source	2628 reflections with *I* > 2σ(*I*)
Mirror monochromator	*R*_int_ = 0.034
Detector resolution: 10.4041 pixels mm^-1^	θ_max_ = 27.6°, θ_min_ = 2.8°
ω scan	*h* = −8→11
Absorption correction: multi-scan (*CrysAlis PRO*; Agilent, 2010)	*k* = −15→10
*T*_min_ = 0.674, *T*_max_ = 1.000	*l* = −14→17
5747 measured reflections	

### Refinement

**Table:**

Refinement on *F*^2^	Primary atom site location: structure-invariant direct methods
Least-squares matrix: full	Secondary atom site location: difference Fourier map
*R*[*F*^2^ > 2σ(*F*^2^)] = 0.039	Hydrogen site location: inferred from neighbouring sites
*wR*(*F*^2^) = 0.105	H atoms treated by a mixture of independent and constrained refinement
*S* = 1.06	*w* = 1/[σ^2^(*F*_o_^2^) + (0.0429*P*)^2^ + 0.1866*P*] where *P* = (*F*_o_^2^ + 2*F*_c_^2^)/3
3314 reflections	(Δ/σ)_max_ = 0.001
198 parameters	Δρ_max_ = 0.54 e Å^−^^3^
2 restraints	Δρ_min_ = −0.52 e Å^−^^3^

### Special details

**Table:**

Geometry. All s.u.'s (except the s.u. in the dihedral angle between two l.s. planes) are estimated using the full covariance matrix. The cell s.u.'s are taken into account individually in the estimation of s.u.'s in distances, angles and torsion angles; correlations between s.u.'s in cell parameters are only used when they are defined by crystal symmetry. An approximate (isotropic) treatment of cell s.u.'s is used for estimating s.u.'s involving l.s. planes.

### Fractional atomic coordinates and isotropic or equivalent isotropic displacement parameters (Å^2^)

**Table:**

	*x*	*y*	*z*	*U*_iso_*/*U*_eq_	
Cu	0.35165 (3)	0.49481 (2)	0.56108 (2)	0.01599 (12)	
O1	0.1585 (2)	0.52540 (15)	0.22861 (14)	0.0225 (4)	
O2	0.2389 (2)	0.50500 (12)	0.42756 (14)	0.0172 (4)	
N1	0.2087 (2)	0.57697 (16)	0.62846 (16)	0.0171 (4)	
N2	0.5049 (3)	0.48980 (18)	0.68604 (18)	0.0185 (5)	
H1N	0.572 (2)	0.5342 (18)	0.670 (2)	0.016 (7)*	
H2N	0.539 (4)	0.4236 (13)	0.679 (3)	0.056 (11)*	
N3	0.4612 (2)	0.36868 (17)	0.50727 (15)	0.0191 (5)	
N4	0.3900 (2)	0.29635 (18)	0.46239 (16)	0.0202 (5)	
N5	0.3257 (3)	0.2250 (2)	0.4194 (2)	0.0331 (6)	
C1	0.1093 (3)	0.5311 (3)	0.1204 (2)	0.0287 (6)	
H1A	0.1670	0.4805	0.0842	0.043*	
H1B	0.1222	0.6058	0.0960	0.043*	
H1C	0.0049	0.5111	0.1066	0.043*	
C2	0.0871 (3)	0.5912 (2)	0.29121 (19)	0.0184 (5)	
C3	−0.0234 (3)	0.6633 (2)	0.2566 (2)	0.0217 (6)	
H3	−0.0517	0.6728	0.1845	0.026*	
C4	−0.0950 (3)	0.7229 (2)	0.3256 (2)	0.0264 (6)	
H4	−0.1717	0.7723	0.3006	0.032*	
C5	−0.0539 (3)	0.7096 (2)	0.4296 (2)	0.0237 (6)	
H5	−0.1035	0.7492	0.4767	0.028*	
C6	0.0620 (3)	0.6374 (2)	0.4675 (2)	0.0184 (5)	
C7	0.1344 (3)	0.5761 (2)	0.39860 (19)	0.0168 (5)	
C8	0.0970 (3)	0.6270 (2)	0.5777 (2)	0.0186 (5)	
H8	0.0307	0.6606	0.6176	0.022*	
C9	0.2165 (3)	0.5744 (2)	0.74125 (19)	0.0190 (5)	
H9A	0.1890	0.5003	0.7621	0.023*	
H9B	0.1427	0.6261	0.7615	0.023*	
C10	0.3675 (3)	0.6033 (2)	0.80083 (19)	0.0183 (5)	
C11	0.4764 (3)	0.5101 (2)	0.7928 (2)	0.0205 (6)	
H11A	0.5707	0.5277	0.8364	0.025*	
H11B	0.4372	0.4425	0.8202	0.025*	
C12	0.3474 (3)	0.6122 (2)	0.9145 (2)	0.0287 (6)	
H12A	0.3124	0.5421	0.9380	0.043*	
H12B	0.2750	0.6693	0.9226	0.043*	
H12C	0.4418	0.6308	0.9558	0.043*	
C13	0.4229 (3)	0.7114 (2)	0.7623 (2)	0.0248 (6)	
H13A	0.5206	0.7277	0.8000	0.037*	
H13B	0.3544	0.7701	0.7737	0.037*	
H13C	0.4294	0.7056	0.6884	0.037*	

### Atomic displacement parameters (Å^2^)

**Table:**

	*U*^11^	*U*^22^	*U*^33^	*U*^12^	*U*^13^	*U*^23^
Cu	0.01581 (19)	0.01867 (19)	0.01328 (18)	0.00302 (11)	0.00137 (14)	−0.00186 (11)
O1	0.0205 (9)	0.0314 (10)	0.0153 (9)	0.0031 (8)	0.0016 (8)	0.0004 (8)
O2	0.0148 (9)	0.0211 (9)	0.0148 (9)	0.0036 (7)	−0.0008 (7)	−0.0003 (7)
N1	0.0155 (10)	0.0178 (10)	0.0185 (10)	−0.0036 (8)	0.0042 (9)	−0.0038 (9)
N2	0.0188 (12)	0.0232 (12)	0.0135 (11)	0.0058 (9)	0.0018 (9)	−0.0015 (9)
N3	0.0215 (11)	0.0181 (10)	0.0179 (11)	0.0026 (9)	0.0035 (9)	−0.0026 (9)
N4	0.0222 (11)	0.0223 (11)	0.0165 (10)	0.0073 (9)	0.0041 (9)	−0.0012 (10)
N5	0.0269 (13)	0.0330 (13)	0.0390 (15)	−0.0034 (11)	0.0035 (12)	−0.0176 (12)
C1	0.0240 (14)	0.0469 (17)	0.0138 (13)	−0.0002 (13)	−0.0018 (11)	0.0008 (13)
C2	0.0137 (12)	0.0193 (12)	0.0220 (13)	−0.0041 (10)	0.0023 (10)	0.0013 (11)
C3	0.0202 (13)	0.0198 (12)	0.0237 (13)	−0.0041 (10)	−0.0019 (11)	0.0063 (11)
C4	0.0195 (13)	0.0213 (13)	0.0360 (16)	0.0019 (11)	−0.0048 (12)	0.0053 (12)
C5	0.0188 (13)	0.0197 (12)	0.0319 (15)	0.0014 (10)	0.0017 (12)	−0.0015 (12)
C6	0.0157 (12)	0.0164 (11)	0.0228 (13)	−0.0020 (10)	0.0019 (11)	−0.0025 (11)
C7	0.0127 (11)	0.0148 (11)	0.0227 (13)	−0.0040 (9)	0.0015 (10)	0.0000 (10)
C8	0.0176 (12)	0.0160 (12)	0.0226 (13)	−0.0023 (10)	0.0046 (11)	−0.0057 (11)
C9	0.0186 (12)	0.0241 (13)	0.0149 (12)	−0.0015 (11)	0.0046 (10)	−0.0028 (11)
C10	0.0204 (13)	0.0192 (12)	0.0155 (12)	−0.0023 (10)	0.0032 (10)	−0.0022 (10)
C11	0.0214 (14)	0.0264 (14)	0.0136 (13)	0.0009 (10)	0.0021 (11)	0.0006 (10)
C12	0.0289 (15)	0.0392 (16)	0.0190 (13)	−0.0011 (13)	0.0071 (12)	−0.0093 (13)
C13	0.0234 (14)	0.0208 (13)	0.0303 (15)	−0.0049 (11)	0.0039 (12)	−0.0052 (12)

### Geometric parameters (Å, º)

**Table:**

Cu—O2	1.9047 (18)	C4—C5	1.370 (4)
Cu—N1	1.960 (2)	C4—H4	0.9500
Cu—N2	2.001 (2)	C5—C6	1.417 (4)
Cu—N3	2.023 (2)	C5—H5	0.9500
Cu—N3^i^	2.641 (2)	C6—C7	1.410 (3)
O1—C2	1.380 (3)	C6—C8	1.440 (3)
O1—C1	1.427 (3)	C8—H8	0.9500
O2—C7	1.310 (3)	C9—C10	1.532 (3)
N1—C8	1.293 (3)	C9—H9A	0.9900
N1—C9	1.469 (3)	C9—H9B	0.9900
N2—C11	1.480 (3)	C10—C13	1.528 (3)
N2—H1N	0.869 (10)	C10—C11	1.529 (4)
N2—H2N	0.877 (10)	C10—C12	1.530 (3)
N3—N4	1.203 (3)	C11—H11A	0.9900
N4—N5	1.154 (3)	C11—H11B	0.9900
C1—H1A	0.9800	C12—H12A	0.9800
C1—H1B	0.9800	C12—H12B	0.9800
C1—H1C	0.9800	C12—H12C	0.9800
C2—C3	1.371 (3)	C13—H13A	0.9800
C2—C7	1.424 (3)	C13—H13B	0.9800
C3—C4	1.397 (4)	C13—H13C	0.9800
C3—H3	0.9500		
			
O2—Cu—N1	93.97 (8)	C6—C5—H5	119.7
O2—Cu—N2	168.34 (9)	C7—C6—C5	120.4 (2)
N1—Cu—N2	94.85 (9)	C7—C6—C8	122.5 (2)
O2—Cu—N3	87.81 (8)	C5—C6—C8	117.1 (2)
N1—Cu—N3	161.12 (9)	O2—C7—C6	124.0 (2)
N2—Cu—N3	86.30 (9)	O2—C7—C2	118.7 (2)
O2—Cu—N3^i^	86.64 (7)	C6—C7—C2	117.3 (2)
N1—Cu—N3^i^	109.71 (7)	N1—C8—C6	127.2 (2)
N2—Cu—N3^i^	83.22 (8)	N1—C8—H8	116.4
N3—Cu—N3^i^	89.15 (8)	C6—C8—H8	116.4
C2—O1—C1	116.9 (2)	N1—C9—C10	114.7 (2)
C7—O2—Cu	126.05 (16)	N1—C9—H9A	108.6
C8—N1—C9	116.6 (2)	C10—C9—H9A	108.6
C8—N1—Cu	122.94 (17)	N1—C9—H9B	108.6
C9—N1—Cu	120.14 (16)	C10—C9—H9B	108.6
C11—N2—Cu	124.71 (18)	H9A—C9—H9B	107.6
C11—N2—H1N	110.5 (18)	C13—C10—C11	111.8 (2)
Cu—N2—H1N	102.8 (18)	C13—C10—C12	110.6 (2)
C11—N2—H2N	111 (2)	C11—C10—C12	106.9 (2)
Cu—N2—H2N	99 (2)	C13—C10—C9	110.5 (2)
H1N—N2—H2N	106 (3)	C11—C10—C9	110.2 (2)
N4—N3—Cu	117.98 (17)	C12—C10—C9	106.6 (2)
N5—N4—N3	177.8 (3)	N2—C11—C10	113.3 (2)
O1—C1—H1A	109.5	N2—C11—H11A	108.9
O1—C1—H1B	109.5	C10—C11—H11A	108.9
H1A—C1—H1B	109.5	N2—C11—H11B	108.9
O1—C1—H1C	109.5	C10—C11—H11B	108.9
H1A—C1—H1C	109.5	H11A—C11—H11B	107.7
H1B—C1—H1C	109.5	C10—C12—H12A	109.5
C3—C2—O1	124.8 (2)	C10—C12—H12B	109.5
C3—C2—C7	121.1 (2)	H12A—C12—H12B	109.5
O1—C2—C7	114.1 (2)	C10—C12—H12C	109.5
C2—C3—C4	121.1 (2)	H12A—C12—H12C	109.5
C2—C3—H3	119.5	H12B—C12—H12C	109.5
C4—C3—H3	119.5	C10—C13—H13A	109.5
C5—C4—C3	119.5 (2)	C10—C13—H13B	109.5
C5—C4—H4	120.2	H13A—C13—H13B	109.5
C3—C4—H4	120.2	C10—C13—H13C	109.5
C4—C5—C6	120.6 (3)	H13A—C13—H13C	109.5
C4—C5—H5	119.7	H13B—C13—H13C	109.5
			
N1—Cu—O2—C7	19.51 (19)	C4—C5—C6—C7	−1.5 (4)
N2—Cu—O2—C7	−119.6 (4)	C4—C5—C6—C8	−179.2 (2)
N3—Cu—O2—C7	−179.31 (19)	Cu—O2—C7—C6	−17.6 (3)
N3^i^—Cu—O2—C7	−90.04 (19)	Cu—O2—C7—C2	164.15 (17)
O2—Cu—N1—C8	−8.9 (2)	C5—C6—C7—O2	−177.6 (2)
N2—Cu—N1—C8	163.5 (2)	C8—C6—C7—O2	−0.1 (4)
N3—Cu—N1—C8	−103.8 (3)	C5—C6—C7—C2	0.7 (3)
N3^i^—Cu—N1—C8	78.9 (2)	C8—C6—C7—C2	178.2 (2)
O2—Cu—N1—C9	164.68 (17)	C3—C2—C7—O2	179.0 (2)
N2—Cu—N1—C9	−22.95 (18)	O1—C2—C7—O2	0.9 (3)
N3—Cu—N1—C9	69.8 (3)	C3—C2—C7—C6	0.6 (3)
N3^i^—Cu—N1—C9	−107.47 (17)	O1—C2—C7—C6	−177.5 (2)
O2—Cu—N2—C11	156.8 (3)	C9—N1—C8—C6	−177.6 (2)
N1—Cu—N2—C11	17.8 (2)	Cu—N1—C8—C6	−3.8 (4)
N3—Cu—N2—C11	−143.3 (2)	C7—C6—C8—N1	11.6 (4)
N3^i^—Cu—N2—C11	127.1 (2)	C5—C6—C8—N1	−170.8 (2)
O2—Cu—N3—N4	−49.87 (19)	C8—N1—C9—C10	−133.4 (2)
N1—Cu—N3—N4	46.0 (4)	Cu—N1—C9—C10	52.6 (3)
N2—Cu—N3—N4	140.2 (2)	N1—C9—C10—C13	51.7 (3)
N3^i^—Cu—N3—N4	−136.5 (2)	N1—C9—C10—C11	−72.3 (3)
C1—O1—C2—C3	−2.2 (4)	N1—C9—C10—C12	172.0 (2)
C1—O1—C2—C7	175.8 (2)	Cu—N2—C11—C10	−39.6 (3)
O1—C2—C3—C4	176.8 (2)	C13—C10—C11—N2	−60.1 (3)
C7—C2—C3—C4	−1.1 (4)	C12—C10—C11—N2	178.8 (2)
C2—C3—C4—C5	0.3 (4)	C9—C10—C11—N2	63.3 (3)
C3—C4—C5—C6	1.0 (4)		

Symmetry code: (i) −*x*+1, −*y*+1, −*z*+1.

### Hydrogen-bond geometry (Å, º)

**Table:**

*D*—H···*A*	*D*—H	H···*A*	*D*···*A*	*D*—H···*A*
N2—H1*N*···O2^i^	0.87 (1)	2.35 (2)	2.956 (3)	127 (2)
N2—H2*N*···N3	0.88 (1)	2.36 (3)	2.752 (3)	107 (3)

Symmetry code: (i) −*x*+1, −*y*+1, −*z*+1.
